# Definición diagnóstica en una familia con *malattia leventinese* en Colombia

**DOI:** 10.7705/biomedica.5604

**Published:** 2021-09-22

**Authors:** Nancy Gelvez, Paula Hurtado-Villa, Silvia Flórez, Anne Charlotte Brieke, Francisco Rodríguez, Ana María Bertolotto, Martha L. Tamayo

**Affiliations:** 1 Instituto de Genética Humana, Pontificia Universidad Javeriana, Bogotá D.C., Colombia Pontificia Universidad Javeriana Instituto de Genética Humana Pontificia Universidad Javeriana BogotáD.C Colombia; 2 Facultad de Ciencias de la Salud, Pontificia Universidad Javeriana, Cali, Colombia Pontificia Universidad Javeriana Facultad de Ciencias de la Salud Pontificia Universidad Javeriana Cali Colombia; 3 Independiente, Cúcuta, Colombia Independiente Cúcuta Colombia; 4 Fundación Oftalmológica Nacional, Escuela de Medicina y Ciencias de la Salud, Universidad del Rosario, Bogotá D.C., Colombia Universidad del Rosario Fundación Oftalmológica Nacional Escuela de Medicina y Ciencias de la Salud Universidad del Rosario BogotáD.C Colombia; 5 Departamento de Pediatría, Facultad de Medicina, Pontificia Universidad Javeriana, Bogotá D.C., Colombia Pontificia Universidad Javeriana Departamento de Pediatría Facultad de Medicina Pontificia Universidad Javeriana BogotáD.C Colombia; 6 Servicio de Pediatría, Hospital Universitario San Ignacio, Bogotá D.C., Colombia Hospital Universitario San Ignacio BogotáD.C Colombia

**Keywords:** distrofias retinianas, epitelio pigmentario de la retina, degeneración macular, retina, Retinal dystrophies, retinal pigment epithelium, macular degeneration, retina

## Abstract

La *malattia leventinese* es una enfermedad hereditaria autosómica dominante, cuyos síntomas se inician entre la segunda y la cuarta décadas de la vida. Se caracteriza por la aparición de drusas localizadas entre el epitelio pigmentario de la retina y la membrana de Bruch; suele reducir la visión drásticamente y progresar a ceguera. La variante patogénica p.Arg345Trp en el gen *EFEMP1* se ha asociado con esta enfermedad. Se presenta aquí la caracterización clínica y molecular de una familia con *malattia leventinese* mediante un manejo integral que involucró a oftalmólogos, pediatras y genetistas, lo que es de gran importancia, ya que el fenotipo de esta enfermedad suele confundirse con la degeneración macular. A todos los individuos de la familia se les hizo la evaluación oftalmológica con imágenes diagnósticas de retina y extracción de ADN a partir de una muestra de sangre periférica. Todos los exones del gen *EFEMP1* se amplificaron y secuenciaron. La variante patogénica p.Arg345Trp se identificó en los individuos afectados. Este es el primer reporte de *malattia leventinese* en una familia con la variante patogénica p.Arg345Trp en Colombia. El diagnóstico molecular de las distrofias retinianas es fundamental para diferenciar este tipo de enfermedades.

## Abstract (en)

The *malattia leventinese* is an autosomal dominant inherited disease whose symptoms appear between the second and fourth decades of life. It is characterized by the appearance of drusen located between the retinal pigment epithelium and the Bruch membrane. It is usually associated with low vision and may progress to blindness. The pathogenic variant p.Arg345Trp in the *EFEMP1* gene has been associated with this disease. We characterized clinically and molecularly a family with *malattia leventinese* using a comprehensive approach that involved ophthalmologists, pediatricians, and geneticists. This approach is of great importance since the phenotype of this disease is often confused with macular degeneration. All family members underwent ophthalmological evaluation and DNA extraction from a peripheral blood sample. All exons of the *EFEMP1* gene were amplified and sequenced. The pathogenic variant p.Arg345Trp was identified in affected individuals in this family.

This is the first report of *malattia leventinese* in a family with the p.Arg345Trp pathogenic variant in Colombia. The molecular diagnosis of retinal dystrophies is essential to differentiate this type of pathology.

La *malattia leventinese* es una rara enfermedad degenerativa de herencia autosómica dominante que afecta la mácula ^1^^-^^3^ y causa pérdida grave de la visión o ceguera legal ^4^. También se la conoce como distrofia retiniana en panal de abejas de Doyne.

La enfermedad generalmente se manifiesta entre los 20 y los 40 años de edad, pero puede aparecer en la niñez o en la tercera edad ^2^. Un rasgo característico temprano de la *malattia leventinese* es la presencia en distribución radial de unos depósitos extracelulares denominados *drusas,* que suelen tener aspecto de "panal de abejas" y que se acumulan entre el epitelio pigmentario de la retina y la membrana de Bruch ^1^^,^^2^. En una etapa más tardía de la enfermedad, se presenta una variedad de características clínicas e histopatológicas, incluidos la atrofia geográfica, los cambios en la pigmentación y la nueva irrigación coroidea, los cuales pueden llevar a disminución de la agudeza visual ^2^.

La *malattia leventinese* fue descrita por primera vez en individuos originarios del valle Leventina, en el sur de Suiza, de ahí su nombre ^5^. Hasta el momento, solo se ha reportado una variante patogénica en el gen *EFEMP1* como causante de la enfermedad, la c.1033C>T/p.Arg345Trp ^1^^,^^6^^,^^7^, la cual se ha detectado en población sueca, británica, australiana, norteamericana, japonesa y coreana ^8^.

Las drusas típicas de esta condición son acumulaciones de material extracelular, localzadas inmediatamente debajo del epitelio pigmentario de la retina ^9^, las cuales pueden ser una manifestación del proceso normal de envejecimiento o representar un signo importante asociado con enfermedades de la retina ^10^. La degeneración macular relacionada con la edad se caracteriza actualmente por la presencia de drusas y muchos fenotipos de drusas retinianas se agrupan bajo esta denominación. Esta es una enfermedad heterogénea y se han identificado hasta 34 *loci* diferentes ^11^. Son pocos los reportes relacionados con la *malattia leventinense,* lo que hace interesante su estudio y diferenciación diagnóstica con respecto a la degeneración macular relacionada con la edad.

En el presente estudio, se hicieron la evaluación oftalmológica completa y el estudio molecular del gen *EFEMP1* en una familia colombiana con tres individuos afectados.

## Población de estudio

**Figura 1:**
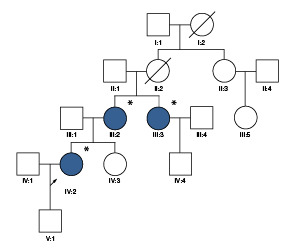
Árbol genealógico de una familia colombiana con *malattia leventinese*

Se incluyeron cinco individuos: tres mujeres afectadas y dos individuos sanos. Esta familia es natural de Barranquilla y en la consulta de genética médica se logró determinar que tenía ancestros del sur de Suiza (figura 1).

## Evaluación clínica

Todos los individuos fueron evaluados por un oftalmólogo especializado en retina y vítreo. Se les practicaron exámenes paraclinicos a dos de las tres mujeres afectadas (figura 1), pero en la tercera no fue posible hacerlo. En el caso de la paciente IV:2, estos se hicieron a los 27 años de edad y, en la paciente III:2, a los 41 años. Los exámenes incluyeron campo visual, angiografía con fluoresceína y electrorretinograma. El análisis molecular se realizó en los laboratorios del Instituto de Genética Humana de la Pontificia Universidad Javeriana Bogotá.

## Análisis del gen *EFEMP1*

Se tomaron muestras de sangre periférica a los cinco individuos incluidos en el estudio. El ADN se extrajo mediante la técnica de fenol-cloroformo y se hicieron análisis de secuenciación bidireccional de Sanger de los 12 exones del gen *EFEMP1.*

Las condiciones de temperatura fueron las siguientes: 95 °C durante 10 segundos, 50 °C durante 5 segundos y 60 °C durante 4 minutos para 35 ciclos. La precipitación de la reacción de secuenciación se hizo con el método de etanol. Las secuencias se analizaron con un equipo ABI-PRISM 3100-Avant™ (Applied Biosystems) usando el estuche de secuenciación BigDye Terminator™, versión 3.1 (Applied Biosystems). Por último, se analizaron las secuencias con el programa SeqScape™ (Applied Biosystems). El procedimiento utilizado para la secuenciación fue el siguiente: una vez obtenido el producto de PCR, se purificó y se hizo la reacción de secuenciación con 2 μl de BigDye, 2 μl de solución tampón 5X, 1 μl de iniciador y 5 μl del producto purificado.

La secuencia consenso utilizada para el gen *EFEMP1* fue la ENST00000355426.8, obtenida en https://www.ensembl.org/Homo_sapiens/Transcript/Exons?db=core;g=ENSG00000115380;r=2:55865967-55924139;t=ENST00000355426.

## Consideraciones éticas

Todos los individuos incluidos firmaron el consentimiento informado, aceptando la toma de muestras y la utilización de los datos de la historia clínica, así como su publicación. El estudio fue aprobado por el Comité de Ética de la Pontificia Universidad Javeriana. El protocolo se apega a la normatividad vigente contemplada en la Declaración de Helsinki de Fortaleza (Brasil) 2013 y la Resolución 8430 de 1993 del Ministerio de Salud de Colombia.

## Hallazgos clínicos

**Figura 2:**
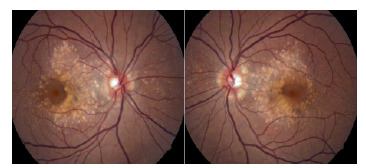
Foto del fondo de ojo de la paciente IV:2. En la angiografía con fluoresceína se aprecia el patrón de las drusas.

Los exámenes paraclínicos se hicieron en dos de las tres mujeres afectadas de la familia (figura 1, cuadro 1). La paciente IV:2 fue valorada por presentar nictalopía como primer síntoma; refirió que su madre y una tía presentaban la enfermedad macular (III:2 y III:3). La mujer tenía alteración bilateral del epitelio pigmentario de la retina, con pigmentación fina, adelgazamiento retiniano macular, visualización de los vasos coroideos y drusas alrededor de la mácula (figura 2). Las alteraciones detectadas eran sugestivas de tres diagnósticos: a) drusas laminares hereditarias, b) retinitis *punctata albecens,* una variante de la retinosis pigmentosa, o c) retina pecosa (grupo de enfermedades caracterizado por múltiples lesiones blancoamarillentas en el fondo de ojo *(fundus albipunctatus, fundus flavimaculatus,* drusas familiares y retina en "flecos" de Kandori). El seguimiento del caso evidenció que la enfermedad progresaba y se asociaba clínicamente con disminución de la visión.

**Cuadro:** 1. Resultados de los exámenes clínicos y paraclínicos de los individuos afectados

	Paciente IV:2	Paciente III:2
Edad (anos)	27	41
Agudeza visual	OD: 20/25; OI: 20/25	OD: 20/60; OI: 20/25
Electrorretinograma	Estimulación fotópica y escotopica normal en ambos ojos	Estimulación fotópica y escotopica disminuida en ambos ojos
Angiografía con Fluoresceína	Múltiples puntos amarillentos hiperfluorescentes en patrón radiado sugestivos de drusas en polo posterior en ambos ojos	Región hiperfluorescente en el área de la fóvea que alterna con puntos hiperfluorescentes alrededor en ambos ojos
Campimetría	Defecto peripapilar asociado con defecto macular incipiente en ambos ojos	OD: prueba de escasa confiabilidad por numerosas pérdidas de fijación. Los cambios sugieren disminución generalizada de la sensibilidad asociada con defecto temporal superior profundo. OI: escasa confiabilidad por numerosas pérdidas de fijación. Los cambios sugieren disminución generalizada de la sensibilidad asociada con defecto temporal superior profundo y defecto paracentral inferior moderado.

OD: ojo derecho; Ol: ojo izquierdo

La paciente III:2 presentaba disminución progresiva de la visión desde los 34 años. El diagnóstico inicial fue de *fundus flavimaculatus,* ya que en la angiografía que le practicaron a esa edad se reportó hiperfluorescencia del área macular con acumulaciones pigmentarias localizadas, que parecían corresponder a una degeneración macular relacionada con la edad y probablemente asociada con un *fundus albipuntatus.* Posteriormente, a la edad de 41 años, recibió un segundo diagnóstico posible, el de distrofia de retina (enfermedad de Stargardt) (cuadro 1) y, a los 48 años, en otra angiografía con fluoresceína, se evidenció una hiperfluorescencia moteada en toda el área macular que permaneció estable a lo largo de las fases de estudio, así como una hiperfluorescencia relativa del nervio óptico en ambos ojos.

A la paciente lll:3, afectada también con disminución de la agudeza visual, no fue posible practicarle los exámenes paraclínicos. Sin embargo, en el examen de fondo de ojo se observaron lesiones amarillentas centrales en el área macular y otras con distorsión del área de la fóvea alternando con áreas de hiperpigmentación.

Los otros dos individuos incluidos en el estudio (IV:3 y IV:4) no presentaban disminución de la agudeza visual y en el examen del fondo de ojo no se reportaron alteraciones evidentes. Cabe destacar que el hijo de la paciente IV:2 era un menor de edad asintomático, razón por la cual no se le tomó muestra para el estudio molecular. En el examen del oftalmólogo pediatra, no se reportaron alteraciones en la retina.

## Análisis molecular del gen *EFEMP1*

**Figura 3:**
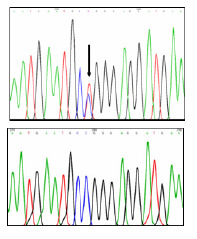
Electroferograma de la variante patogénica p.Arg345Trp en el gen *EFEMP1.* Arriba: secuencia con variante patogénica. Abajo: secuencia normal

En los individuos III:2, III:3 y IV:2, se detectó la variante patogénica p.Arg345Trp en el gen *EFEMP1* en estado heterocigoto (figura 3). Esta variante no se halló en los individuos sanos IV:3 y IV:4.

## Discusión

La correlación entre los hallazgos clínicos de las tres mujeres afectadas, los resultados del estudio molecular y el antecedente familiar de ancestros originarios del valle del Leventino en el sur de Suiza, llevó al diagnóstico de enfermedad del valle Leventina *(malattia leventinese),* degeneración macular que conlleva disminución de la agudeza visual a edades tempranas, caracterizada por la presencia de drusas pequeñas de disposición radial en la periferia y drusas grandes confluyentes en el centro de la mácula, también conocidas como drusas dominantes o familiares.

La enfermedad fue inicialmente descrita en 1925 por Vogt en pacientes del valle del Leventino en Suiza. Lo usual es que se inicie de forma simétrica entre la segunda y la cuarta décadas de la vida, en algunos casos, con disminución de la agudeza visual o metamorfopsias; no obstante, muchas otras personas pueden permanecer asintomáticas hasta edades más avanzadas.

El principal diagnóstico diferencial por fenotipo es la distrofia retiniana en panal de abejas de Doyne ^3^ y, aunque algunos autores han planteado que probablemente se trate de la misma enfermedad ^2^, otros afirman que son diferentes y que se distinguen por la variable disposición de las drusas; lo clásico en la *malattia leventinese* es que se sitúen en un patrón radial o radio de rueda ^5^. Otro diagnóstico diferencial es la degeneración macular relacionada con la edad, pero debe tenerse en cuenta que la principal diferencia está en la edad de inicio de la enfermedad, pues en la *malattia leventinese* generalmente se inicia entre la segunda y la cuarta décadas de la vida, en tanto que la degeneracion macular aparece en edades más avanzadas ^2^^,^^4^.

La variante patogénica p.Arg345Trp, identificada en esta familia, es la única asociada con la enfermedad hasta el momento en diferentes poblaciones ^1^^,^^5^^-^^8^. En los estudios en pacientes de diferentes etnias, solo una vez se logró determinar esta variante en un ancestro común de origen suizo ^1^.

La edad de inicio de la enfermedad coincide con lo reportado por otros autores, ya que las mujeres afectadas manifestaron los primeros síntomas a los 27 y los 28 años. Como ya se dijo, los síntomas generalmente aparecen entre los 20 y los 40 años ^12^; sin embargo, en estas pacientes el diagnóstico clínico no se estableció a la edad de inicio de los síntomas, sino más tarde. Los hallazgos característicos incluyen drusas de inicio temprano en el polo posterior, a menudo dispuestas en una configuración radial, así como depósitos en el margen del disco óptico, drusas nasales en el disco óptico, atrofia del epitelio pigmentario de la retina en la mácula y la formación de membranas neovasculares subretinianas ^13^.

En la paciente III:2, los exámenes paraclínicos se practicaron de forma tardía, a pesar de que los síntomas oculares comenzaron a los 28 años, pero solo se detectaron cambios pigmentarios retinianos inespecíficos y muy avanzados que no permitieron hacer un diagnóstico; algo similar ocurrió con su hermana (paciente III:3). Por el contrario, al consultar al oftalmólogo especialista en retina y vítreo, la paciente IV:2, cuyos síntomas se iniciaron a los 27 años, fue remitida a genética con sospecha de una alteración retiniana hereditaria. En la consulta de genética médica, se detectaron los casos de los otros familiares, se identificó el patrón hereditario de la enfermedad y se encontró un ancestro originario del sur de Suiza.

Por último, fue el estudio molecular del gen *EFEMP1* lo que permitió el diagnóstico clínico en esta familia. En el cuadro 1 se presentan los hallazgos de los exámenes paraclínicos de las pacientes IV:2 y III:2. Es importante resaltar que las manifestaciones clínicas son más evidentes y características de la enfermedad en etapa temprana. En la angiografía con fluoresceína de la paciente IV:2, se halló el patrón de drusas (figura 2), en tanto que, en la paciente III:2, no se evidenció una diferenciación de este patrón.

El hecho de que uno o varios individuos de una misma familia consulten muy tardíamente, o lo hagan por separado, lleva a que los servicios de oftalmología general no sospechen la condición hereditaria y los afectados no obtengan un diagnóstico específico en muchos años. Este es un llamado a los oftalmólogos generales y a los especializados en retina y vítreo a indagar más exhaustivamente sobre los antecedentes familiares y a remitir al genetista casos como este, en el que no se había indagado ni preguntado sobre el origen suizo de la familia.

El gen *EFEMP1* codifica para la proteína fibulina-3, una glucoproteína de matriz extracelular. Aunque su función aún no se comprende por completo, hay hallazgos que la involucran en una gran cantidad de procesos fisiopatológicos ^14^. Los datos recopilados sugieren que una mutación en este gen puede inducir la enfermedad mediante un mecanismo de ganancia de función ^1^. Se ha propuesto que la acumulación de fibulina-3 mal plegada en el retículo endoplasmático de las células del epitelio pigmentario retiniano induce al estrés de dicho retículo y la activación de la reacción de proteína desplegada *(unfolded protein response,* UPR) ^15^. Esta reacción es una cascada de señalización celular adaptativa que facilita las vías de eliminación y degradación de las proteínas del retículo endoplasmático ^16^, proceso que podría estimular la aparición de características reconocidas de la *malattia leventinese,* incluida la disfunción del epitelio pigmentario retiniano, la apoptosis, la eventual atrofia y la nueva irrigación coroidea ^15^.

El diagnóstico molecular de las distrofias maculares relacionadas con la edad es cada vez más relevante, pues permite precisar el diagnóstico. Se han descrito distrofias maculares monogénicas que también se caracterizan principalmente por drusas del polo posterior ^13^. El diagnóstico molecular abre la posibilidad de un tratamiento oportuno que permita retrasar la progresión de enfermedades que amenazan la visión, como la *malattia leventinese.*

Además, es importante que los pediatras y los oftalmólogos con subespecialidad en pediatría estén alerta cuando haya menores de edad en familias con una enfermedad autosómica dominante, pues existe una probabilidad del 50 % de heredar el gen afectado. El dilema ético implícito en la decisión de hacer la prueba molecular está entre hacer un diagnóstico precoz que puede beneficiar la calidad de vida del niño y enfrentar las consecuencias muy bien conocidas de hacer un diagnóstico molecular presintomático.

En este caso, se propuso a los padres hacer un seguimiento clínico oftalmológico permanente, de manera que, al momento de evidenciarse el más mínimo síntoma o signo de la enfermedad, se procediera a la prueba molecular a partir de una sospecha diagnóstica específica. De ahí que sea preciso alertar a los especialistas pediátricos a estar atentos ante este tipo de situaciones.

Dado que no ha habido reportes previos de esta enfermedad en una familia colombiana, este estudio brinda una herramienta muy útil para la asesoría genética y una base para futuros estudios relacionados con los diagnósticos diferenciales de la degeneración macular relacionada con la edad.
